# Optimized chaotic Brillouin dynamic grating with filtered optical feedback

**DOI:** 10.1038/s41598-018-19180-w

**Published:** 2018-01-16

**Authors:** Jianzhong Zhang, Zhuping Li, Yuan Wu, Mingjiang Zhang, Yi Liu, Mengwen Li

**Affiliations:** 1 Key Laboratory of Advanced Transducers and Intelligent Control System, Ministry of Education and Shanxi Province, Taiyuan University of Technology, Taiyuan, 030024 People’s Republic of China; 2 Institute of Optoelectronic Engineering, College of Physics and Optoelectronics, Taiyuan University of Technology, Taiyuan, 030024 People’s Republic of China

## Abstract

Chaotic Brillouin dynamic gratings (BDGs) have special advantages such as the creation of single, permanent and localized BDG. However, the periodic signals induced by conventional optical feedback (COF) in chaotic semiconductor lasers can lead to the generation of spurious BDGs, which will limit the application of chaotic BDGs. In this paper, filtered optical feedback (FOF) is proposed to eliminate spurious BDGs. By controlling the spectral width of the optical filter and its detuning from the laser frequency, semiconductor lasers with FOF operate in the suppression region of the time-delay signature, and chaotic outputs serving as pump waves are then utilized to generate the chaotic BDG in a polarization maintaining fiber. Through comparative analysis of the COF and FOF schemes, it has been demonstrated that spurious BDGs are effectively eliminated and that the reflection characterization of the chaotic BDG is improved. The influence of FOF on the reflection and gain spectra of the chaotic BDG is analyzed as well.

## Introduction

Brillouin dynamic gratings (BDGs) have attracted increasing and extensive attention due to their applications in distributed fiber sensing^1^, tunable optical delays^2^, all-optical flip-flops^3^, all-optical signal processing^4^, and high-resolution optical spectrometers^5^. A BDG is generated by the stimulated Brillouin scattering (SBS) interaction between two counter-propagating pump waves in optical fibers. Since the first proof of concept experiment on the BDG in a polarization maintaining fiber (PMF) was reported in 2008^6^, BDGs have been successfully generated in single mode fibers^7,8^, few-mode fibers^9^, photonic crystal fibers^10^ and integrated chips^11^. Meanwhile, the spectral characteristics of BDGs in PMFs have been analyzed in detail^12,13^. In addition, different types of BDGs have been proposed and realized including, for example, phase-shifted BDGs^14^, chirped BDGs^15^ and sampled BDGs^16^.

Distributed measurements of BDGs have been verified utilizing time domain and correlation domain techniques. In a time domain system, the pulse signals are usually utilized as the pump waves to generate a BDG^17,18^. However, the formation of a BDG requires a very short pulse signal with a peak power of several hundred watts. This leads to a complex structure and high cost in the time domain system. In a correlation domain system, continuous waves with frequency modulation by sinusoidal signals^19^ or phase modulation by pseudo-random bit sequences (PRBSs)^20–22^ are employed to produce a BDG. However, autocorrelations of sine-modulated or PRBS-modulated continuous waves have periodic correlation peaks, which give rise to the creation of multiple BDGs. To generate a single BDG in an optical fiber, amplified spontaneous emissions (ASEs) with single correlation peaks can be used as pump waves^23^.

Chaotic lasers are another signal format used to obtain single correlation peaks and have been proven to form single, permanent BDGs in PMFs^24^. Recently, the reflection and gain spectra characteristics of chaotic BDGs have been investigated in detail^25^. However, in the generation of a chaotic BDG, a spurious BDG resulting from the autocorrelation of a weak amplitude periodic signal emerges. The SBS amplification in the spurious BDG contributes an additional noise mechanism, which can largely limit the signal to noise ratio (SNR) of the chaotic Brillouin optical correlation domain analysis (BOCDA) system^26^. Therefore, the elimination of the spurious BDG is critical for applications of chaotic BDGs.

In fact, the essential cause of the spurious BDG generation is that chaotic light from a semiconductor laser with conventional optical feedback (COF) contains a periodic signal induced by the optical round trip in the external cavity feedback. Therefore, the feedback scheme needs to be modified to cancel the external-cavity-induced periodic signal. Filtered optical feedback (FOF) is a simple and controllable feedback mechanism achieved by adjusting the filter’s bandwidth and its detuning from the laser frequency^27^. Under its influence, semiconductor lasers can not only output chaotic signals^28^ but also suppress the feedback time delay signature^29^.

In this paper, a semiconductor laser with FOF is introduced to generate a chaotic BDG, and the time-delay-induced spurious BDG is effectively eliminated. The characteristics of the chaotic BDG are further investigated by comparing the FOF and COF schemes.

## Results and Discussion

**Figure 1:**
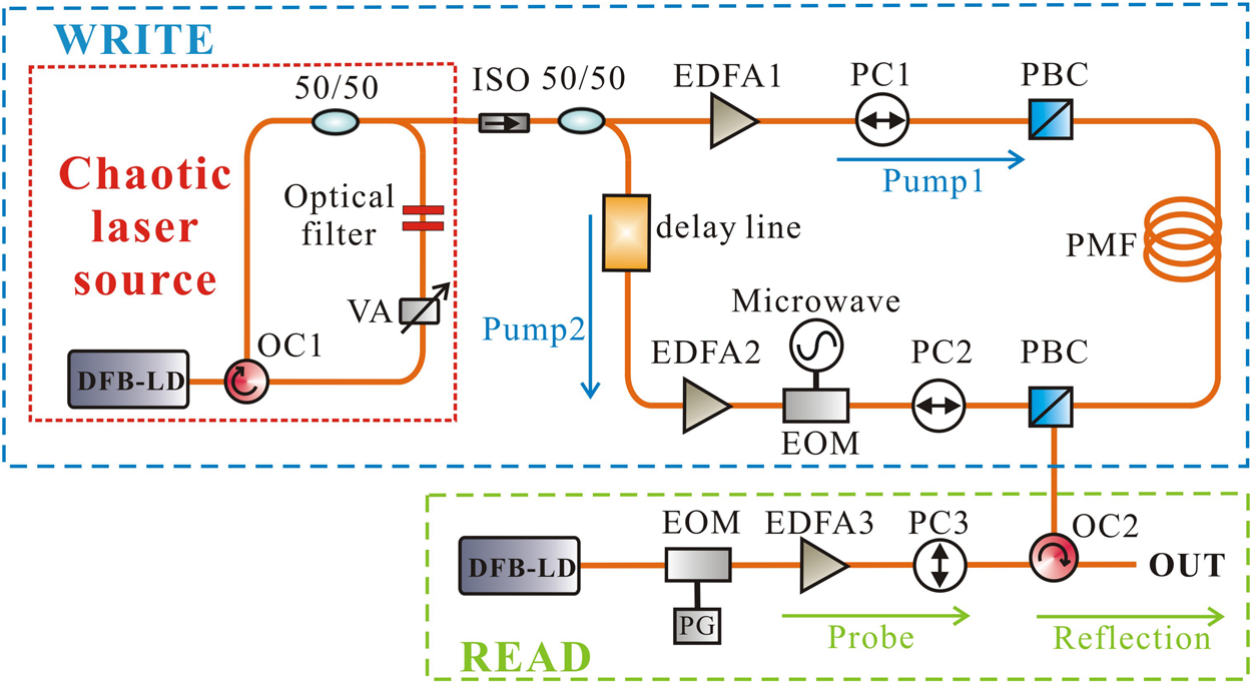
Schematic diagram of the chaotic BDG generation and reading processes. DFB-LD: distributed-feedback laser diode; OC: optical circulator; VA: variable attenuator; ISO: isolator; PC: polarization controller; EDFA: erbium-doped optical fiber amplifier; PBC: polarization beam combiner; PMF: polarization maintaining fiber; EOM: electro-optic modulator; PG: pulse generator.

A schematic diagram of the chaotic BDG generation and reading processes is shown in Fig. 1. The chaotic laser source, which is plotted in the red dashed box in Fig. 1, consists of a distributed-feedback laser diode (DFB-LD) and an external feedback cavity formed by an optical circulator (OC), 3 dB optical coupler (50/50), optical filter, and variable attenuator (VA). Here, a Fabry-Pérot interferometer was used as the optical filter to form the FOF. Under the appropriate feedback condition, the DFB-LD is driven into chaotic oscillation. The output chaotic light is equally divided into two beams by a 50/50 fiber coupler as Pump1 and Pump2. By adjusting the polarization controller (PC) and variable optical delay line, Pump1 and Pump2 are injected simultaneously into the PMF from both ends along the slow axis (x-polarization). To strengthen the generated chaotic BDG at the encounter location, Pump1 and Pump2 satisfy the SBS phase-matching condition^6^, that is, *ν*_pump1_ − *ν*_pump2_ = *ν*_*B*_ (*ν*_*B*_ being the Brillouin frequency shift of the PMF), by adjusting the modulation frequency of Pump2. In the probe path, an electro-optic modulator (EOM) and pulse generator are used to generate the probe pulse. The probe signal is then injected into the PMF along the fast axis (y-polarization) and the injection direction is consistent with Pump2. The reflection of the chaotic BDG will be maximized when the frequency difference Δ_*υBire*_ between the probe and Pump2 meets the phase-matching condition^18^.

First, the time-delay signature of the output chaos from the semiconductor laser with FOF can be effectively suppressed by adjusting the parameters of the optical filter, i.e., the spectral width Λ of the filter and its detuning Δ*ν* from the laser frequency. Generally, many indicators such as the autocorrelation function (ACF), delay mutual information (DMI), filling factor analysis, global nonlinear models with neural networks and local linear models can be utilized to identify the time-delay signature^30^. Here, we adopt the ACF and DMI methods. Both methods search for the maximum correspondence of the pair of time series *P*(*t*) and its delayed series *P*(*t* + Δ*t*) as a function of a time-shift Δ*t*. For a chaotic delay-differential system, the ACF is defined as

1 $$C({\rm{\Delta }}t)=\frac{\langle (P(t+{\rm{\Delta }}t)-\langle P(t)\rangle )(P(t)-\langle P(t)\rangle )\rangle }{{({\langle P(t)-\langle P(t)\rangle \rangle }^{2}{\langle P(t+{\rm{\Delta }}t)-\langle P(t)\rangle \rangle }^{2})}^{1/2}},$$

where *P*(*t*) represents the chaotic time series, Δ*t* is the time shift, and <•> denotes time average. The time delay signature can be retrieved from the peak location of the ACF curve. The DMI between *P*(*t*) and *P*(*t* + Δ*t*) is expressed by

2 $$M({\rm{\Delta }}t)=\sum _{E(t),E(t+{\rm{\Delta }}t)}\rho (P(t),P(t+{\rm{\Delta }}t))\mathrm{log}\,\frac{\rho (P(t),P(t+{\rm{\Delta }}t))}{\rho (P(t))\rho (P(t+{\rm{\Delta }}t))},$$

where *ρ*(*P*(*t*), *P*(*t* + Δ*t*)) is the joint probability density, and *ρ*(*P*(*t*)) and *ρ*(*P*(*t* + Δ*t*)) are the marginal probability densities, respectively. The DMI peak location can also be used to identify the time delay signature.

**Figure 2:**
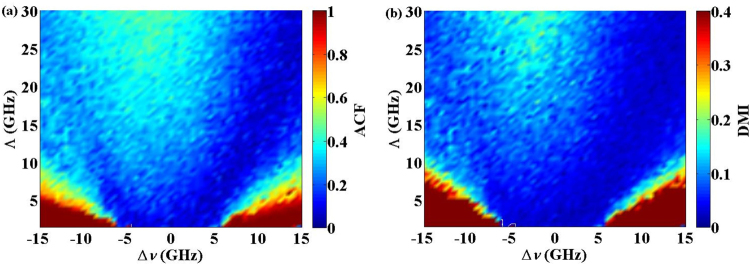
The maps of the time-delay signatures of the output chaos from the semiconductor laser with FOF, where Λ and Δ*ν* vary from 1 GHz to 30 GHz and from −15 GHz to 15 GHz, respectively. The external cavity feedback strength was *κ* = 15% and the pump current *I* = 1.5*I*_th_. (**a**) The distribution of the ACF peak valves at *τ* = 3 ns; (**b**) The distribution of the DMI peak values at *τ* = 3 ns.

Figure 2 illustrates the maps of the time-delay signature of the output chaos from the semiconductor laser with FOF, where Λ and Δν vary from 1 GHz to 30 GHz and from −15 GHz to 15 GHz, respectively. For the results shown in the Fig. 2, the external cavity feedback strength of the semiconductor laser was set to *κ* = 15% and the pump current was *I = *1.5 *I*_th_. Figure 2(a) and (b) correspond to the distributions of the ACF and DMI peak values at the external cavity time-delay *τ* = 3 ns, respectively. From Fig. 2(a), we can see that the dark blue region represents the effective suppression of the time-delay signature with the correlation coefficient of the ACF peak at *τ* = 3 ns, which was less than 0.08. In contrast, the red region indicates that the ACF correlation coefficient at *τ* = 3 ns exceeded 0.9, and the time-delay signatures became very strong. For the distribution of the DMI peak values at *τ* = 3 ns, the suppression region of the time-delay signature was almost consistent with that of the ACF. Therefore, it is convenient to obtain the chaotic signal without an external-cavity-induced periodic signal from the semiconductor laser with FOF by appropriately selecting the parameters of the optical filter.

**Figure 3:**
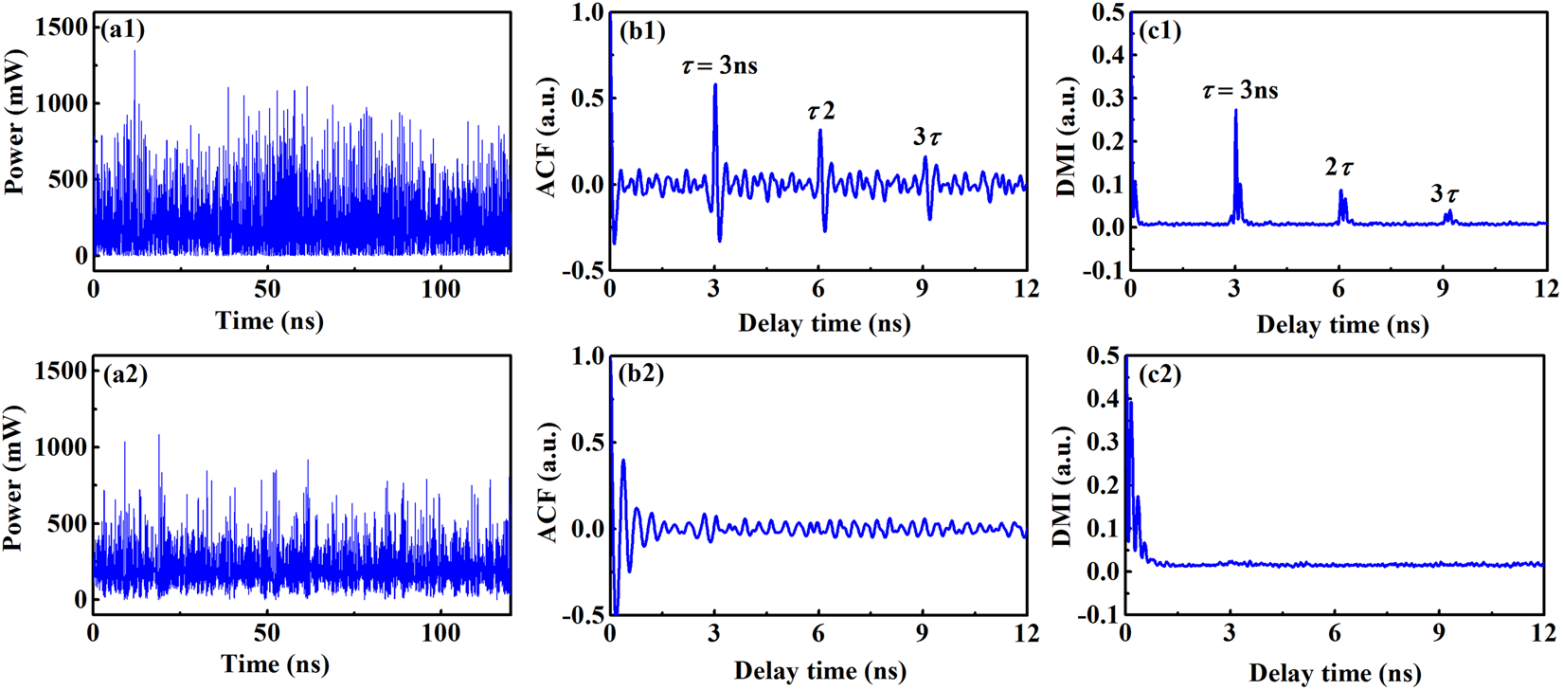
(**a**) Time series, (**b**) ACF curves, and (**c**) DMI curves of the chaotic signals from semiconductor lasers with COF (first row) and FOF (second row), where the external cavity time-delay was *τ* = 3 ns, the external cavity feedback strength was *κ* = 15%, and the pump current was *I* = 1.5*I*_th_. The spectral width of the filter and its detuning from the laser frequency were chosen as Λ = 4 GHz and Δ*ν* = 5.02 GHz, respectively.

To visually display the suppression effect of the time-delay signature, Fig. 3 further presents the time series, ACF curves and DMI curves of the chaotic signals from semiconductor lasers with COF and FOF, respectively. Here, the parameters of the optical filter were arbitrarily chosen from the suppression region of the time-delay signature shown in Fig. 2; as an example, the spectral width of the filter was Λ = 4 GHz, and its detuning from the laser frequency was Δ*ν* = 5.02 GHz. For the COF and FOF schemes, the external parameters of the semiconductor lasers were the same, with the exception of their identical internal parameter; for example, the external cavity time-delay was *τ* = 3 ns, the external cavity feedback strength was *κ* = 15%, and the pump current was *I* = 1.5 *I*_th_. From Fig. 3, we can see that at the locations of the time-delays, (*τ*, 2*τ* and 3*τ*), there were apparent peaks in the ACF and DMI curves for the COF scheme. Conversely, the peaks of the ACF and DMI curves at the time-delay locations completely disappeared for the FOF scheme. The above results again demonstrate that FOF can effectively suppress the time-delay signature by controlling the spectral width of the filter and its detuning from the laser frequency.

### Elimination of the time-delay-induced spurious BDG

The chaotic light signals from the semiconductor lasers with COF and FOF, which are shown in Fig. 3(a1) and (a2), respectively, were then injected into a 1 m PMF to generate the chaotic BDG. The formation of the chaotic BDG was numerically realized according to the equations (5) given in the Methods section below. In fact, the interference of two chaotic pump waves results in a traveling acoustic wave through the mechanism of electrostriction. Because of the photo-elastic effect, the acoustic wave is associated with refractive index variations, and is known as the chaotic BDG. Therefore, the acoustic wave field *Q* in equations (5) can represent the generated chaotic BDG.

**Figure 4:**
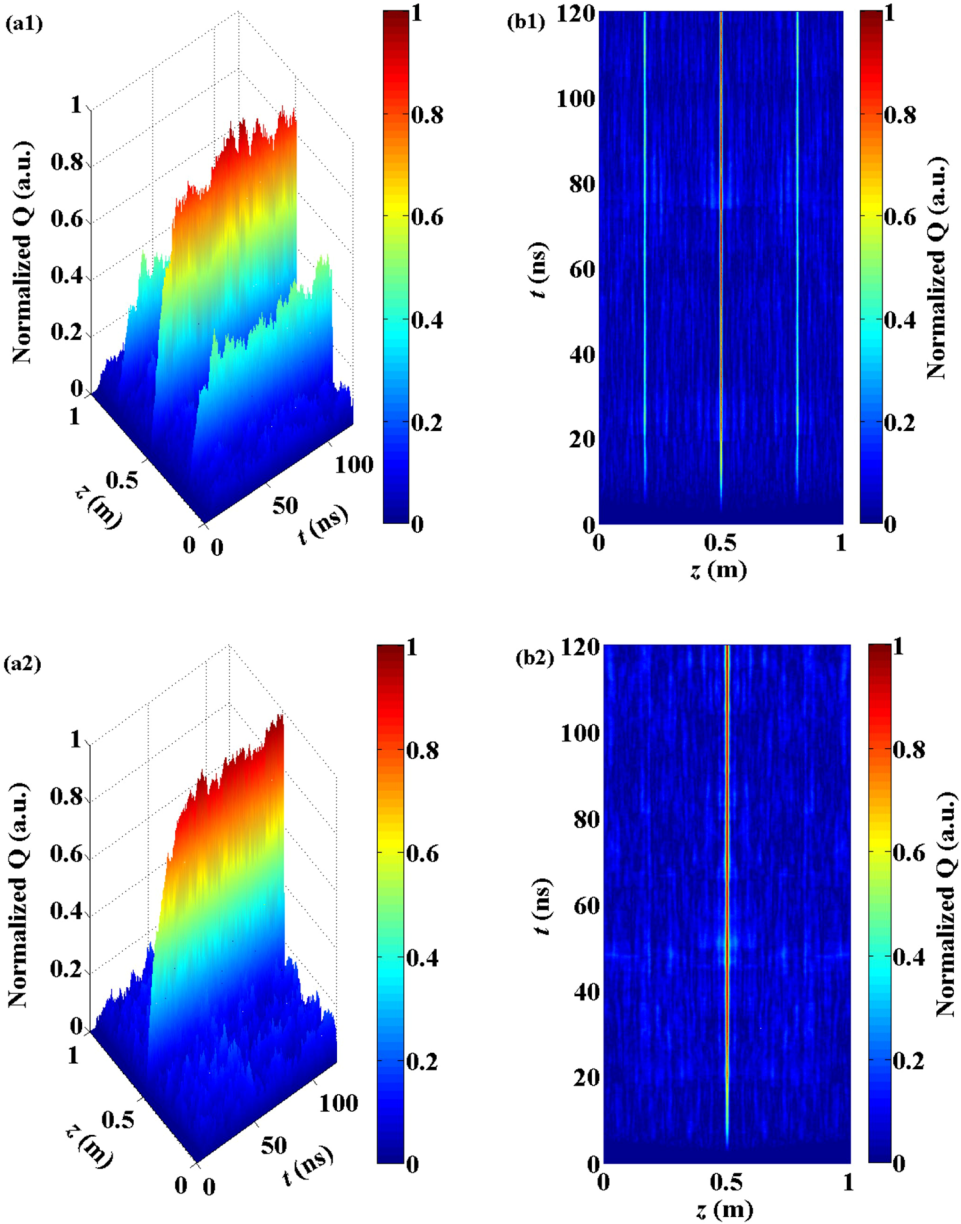
The three-dimensional and two-dimension projection distributions of the acoustic wave field *Q*(*z*, *t*) corresponding to the chaotic BDG. The upper and lower rows represent the distributions for the COF and FOF schemes, respectively.

Figure 4 depicts the three-dimensional and two-dimension projection distributions of the acoustic wave field *Q*(*z*, *t*) in the PMF as a function of time and space. The upper and lower rows in Fig. 4 correspond to the COF and FOF schemes, respectively. For the COF scheme, it can be seen that the two time-delay-induced spurious BDGs were distributed symmetrically on both sides of the chaotic BDG. The distance between the spurious BDG and the chaotic BDG was exactly equal to the external cavity feedback delay of *τ* = 3 ns. However, for the FOF scheme, there was no spurious BDG at the corresponding time-delay locations (0.2 m and 0.8 m) away from the chaotic BDG. This means that in the suppression region of the time-delay signature, the chaotic signals from the semiconductor laser with FOF can produce a chaotic BDG with the elimination of the time-delay-induced spurious BDG.

In addition, we observe that some fluctuant sidelobes remained around the chaotic BDG, although they were relatively small. This is because the amplitude of the chaotic BDG was proportional to the temporal cross correlation between the complex envelopes of the two pump waves. Just when equation (5-5) is analytically solved where the probe and reflected waves are ignored, the corresponding acoustic wave magnitude is given as

3 $$Q(t,z)=\frac{1}{2{\tau }_{B}}{\int }_{0}^{t}\exp (\frac{{t}^{1}-t}{2{\tau }_{B}})A({t}^{1}-\frac{z}{{v}_{g}}){A}^{\ast }[{t}^{1}-\frac{z}{{v}_{g}}+\theta (z)]d{t}^{1},$$

where *A*(*t*) is the complex envelope of the chaotic pump wave, *v*_*g*_ is the group velocity of light in the fiber, and *τ*_*B*_ is the acoustic wave lifetime. The position-dependent temporal offset *θ*(*z*) is defined as *θ*(*z*) = (2*z* − *L*)/*v*_*g*_, where *L* is the fiber length.

The expectation value of the acoustic field magnitude at position *z* (for *t* ≫ *τ*_*B*_) is

4 $$\overline{Q(t,z)}=\frac{1}{2{\tau }_{B}}{\int }_{0}^{t}\exp (\frac{{t}^{1}-t}{2{\tau }_{B}})\overline{A({t}^{1}-\frac{z}{{v}_{g}}){A}^{\ast }[{t}^{1}-\frac{z}{{v}_{g}}+\theta (z)]}d{t}^{1}=c(\theta (z)),$$

where *c*(*θ*(*z*)) is the cross correlation between two chaotic pump waves. At the center (*z* = *L*/2) of the PMF, the chaotic BDG is steadily and permanently generated within the correlation peak width. However, in other locations, *c*(*θ*(*z*)) randomly fluctuates due to the own characteristics of the cross correlation of the chaotic laser. Therefere, some small fluctuant sidelobes emerge around the chaotic BDG. The suppression of those fluctuant sidelobes will be investigated in future work.

**Figure 5:**
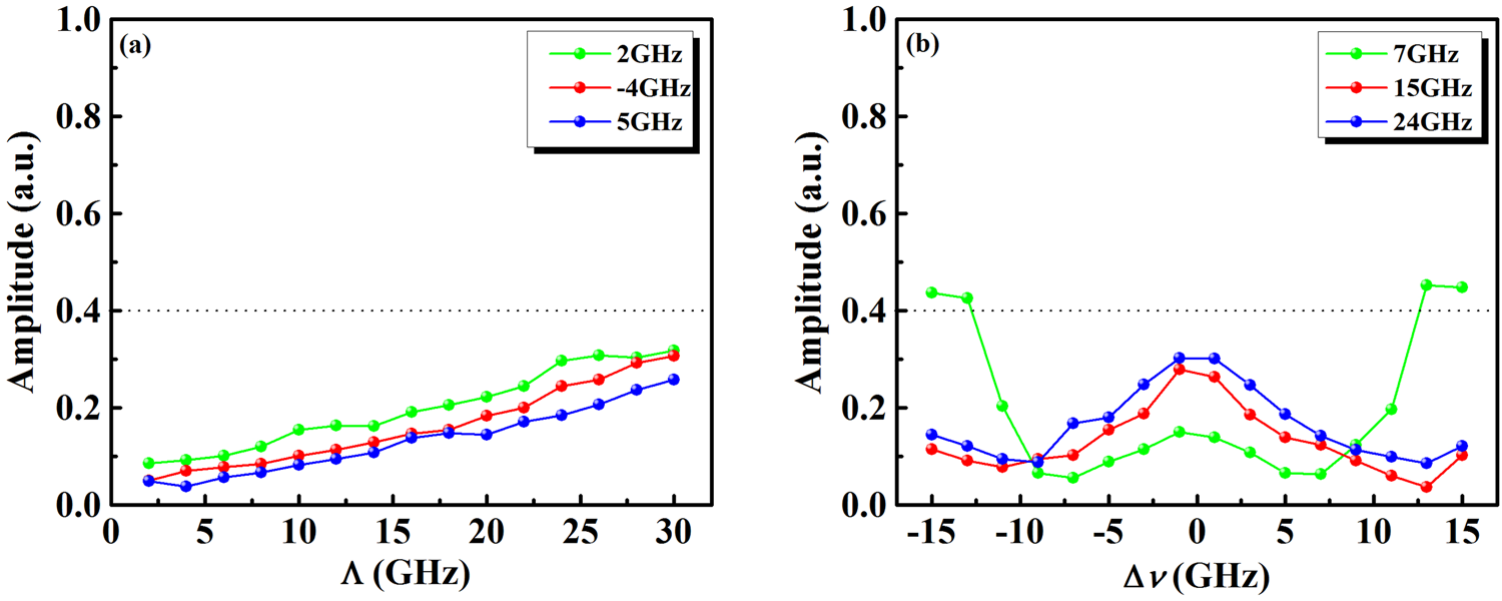
The suppression effect of the spurious BDG versus the filter parameters. (a) The magnitude of the spurious BDG as a function of the spectral width Λ for detunings of Δ*ν* = 2, −4, and 5 GHz, (b) The magnitude of the spurious BDG as a fuction of duning Δ*ν* for spectral widths of Λ = 7, 15, and 24 GHz. For the COF scheme, the amplitude of the original spurious BDG was approximately 0.4.

We further analyzed the suppression effect of the spurious BDG versus the filter parameters. Figure 5(a) presents the amplitudes of the spurious BDG as a function of spectral width Λ under detunings of Δ*ν* = 2 GHz, −4 GHz and 5 GHz. For the COF scheme, the amplitude of the original spurious BDG was approximately 0.4. Obviously, through the use of the FOF, the spurious BDG was effectively suppressed. Within the region of the spectral width below 8 GHz, the amplitude of the spurious BDG is decreased to the level (0.07) of the background fluctuations. Figure 5(b) shows the amplitudes of the spurious BDG as a function of detuning Δ*ν* under spectral widths of Λ = 7 GHz, 15 GHz and 24 GHz. We observe that an optimal detuning parameter exists that can reduce the amplitude of the spurious BDG to its minimum value.

### Reflection characterization improvement for the chaotic BDG

**Figure 6:**
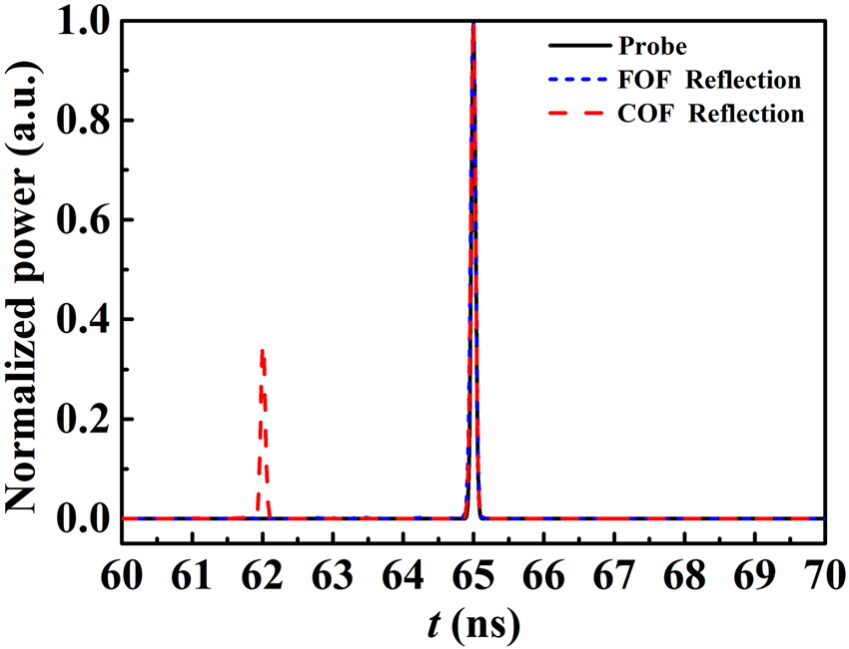
The reflection outputs of the probe pulses by the chaotic BDG for the COF and FOF schemes. The black solid, red dashed and blue short dashed lines refer to the probe pulse, reflection pulse for the COF scheme and reflection pulse for the FOF scheme, respectively.

In what follows, the reflection characterization of the chaotic BDG generated by the FOF scheme is further analyzed using filter parameters of Λ = 4 GHz and Δ*ν* = 5.02 GHz. A probe pulse was injected along the fast axis of the PMF with the same incident direction as Pump2. In the numerical simulation, the probe pulse was Gaussian, i.e., *A*_*p*_(*L*, *t*) = *A* exp(−4ln2*t*^2^/*τ*^2^_FWHM_), where *A* is the amplitude of the probe pulse, and *τ*_FWHM_ is the full width at half maximum (FWHM, 100 ps). The probe pulse is shown as the black solid line in Fig. 6. When a probe pulse is launched onto a chaotic BDG in a PMF, it can be reflected by the chaotic BDG. For comparison, the reflection characterization of the chaotic BDG for the COF scheme is presented as well. The reflection pulses produced by the chaotic BDG for the COF and FOF schemes are illustrated as the red dashed and the blue short dashed lines in Fig. 6, respectively. It can be seen that the reflection pulse was completely coincident with the incident probe pulse. For the COF scheme, at the 3 ns delay-advancement location of the reflection pulse, a small ghost pulse appeared due to the reflection of the spurious BDG. Conversely, for the FOF scheme, the ghost pulse had been completely eliminated through the utilization of the FOF. By calculation, the peak valve of the sidelobe ghost pulse had been reduced by about 25 dB. This means that the reflection characterization of the chaotic BDG can be effectively improved by the FOF.

**Figure 7:**
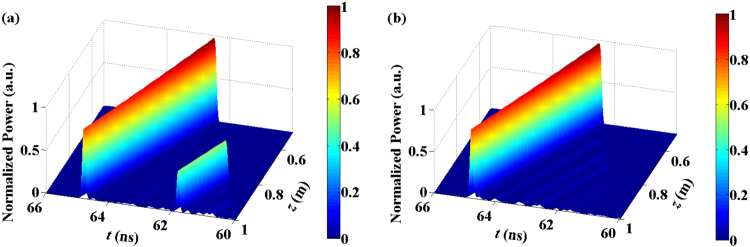
The transmission of the reflection pulse after the probe pulse was reflected by the chaotic BDG of the COF (**a**) and FOF (**b**) schemes, respectively.

Figure 7 further shows the reflected pulse transmission in the PMF after the probe pulse was reflected by the chaotic BDG. Figure 7(a) and (b) correspond to the COF and FOF schemes, respectively. We can see that the time-delay-induced spurious BDG indeed had a detrimental effect on the reflection characterization of the chaotic BDG. With the elimination of the spurious BDG by the FOF, the ghost pulse disappeared.

### Influence of the FOF on the reflection and gain spectra

**Figure 8:**
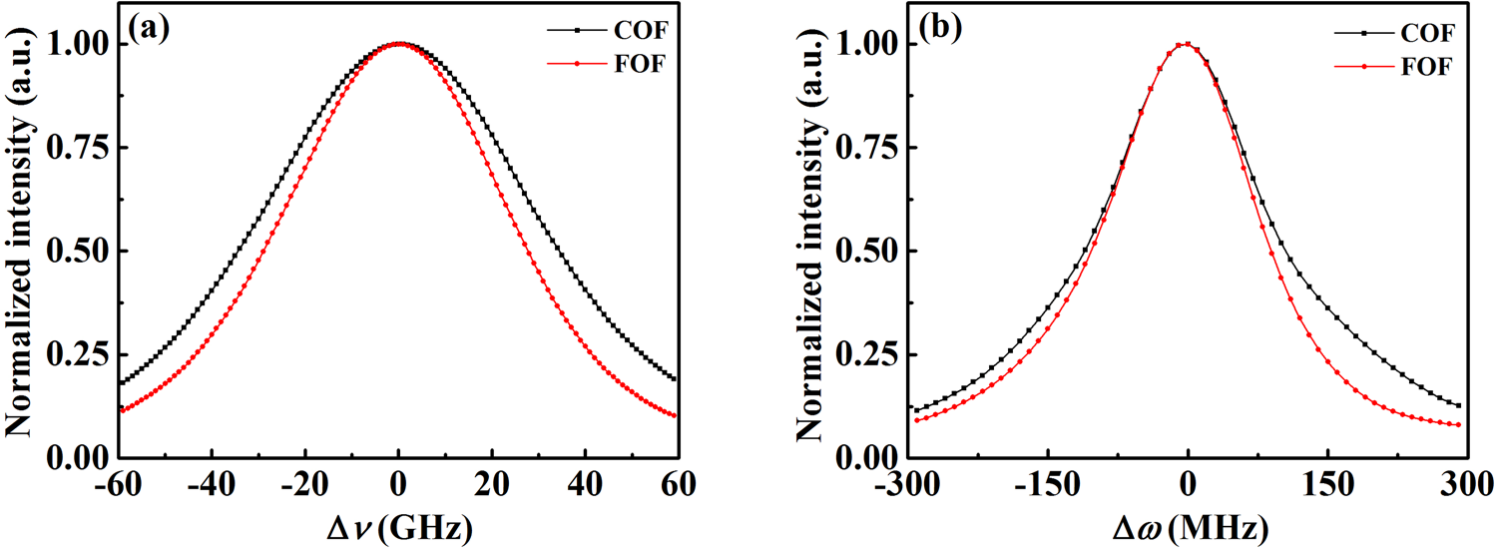
The reflection spectra (**a**) and the gain spectra (**b**) of the chaotic BDGs for the COF and FOF schemes. The red and black lines with markers denote the FOF and COF schemes, respectively.

As demonstrated above, the FOF is a simple and effective feedback control method to suppress the time-delay signature of chaotic signals and to further generate chaotic BDGs that do not contain time-delay-induced spurious BDG. We analyzed the influence of the FOF on the reflection and gain spectra of the generated chaotic BDG by comparing the COF and FOF schemes. Figure 8(a) illustrates the reflection spectra of the chaotic BDG before and after the spurious BDG was eliminated by the FOF. The FWHMs of the reflection spectra of the COF and FOF chaotic BDGs were approximately 68.8 GHz and 56.7 GHz, respectively. Compared with the COF chaotic BDG, the reflection spectrum of the FOF chaotic BDG was narrowed because the reflection spectrum bandwidth of the BDG was inversely proportional to its effective grating length, as has been demonstrated in several experiments^7,12^. The chaotic BDG is formed within the coherence length of the chaotic optical source. Thus, the reflection spectrum bandwidth of the chaotic BDG is ultimately proportional to the optical spectrum bandwidth of the chaotic optical source. The bandwidths of the chaotic light signals from the semiconductor lasers with COF and FOF were further measured to be approximately 15 GHz and 8 GHz, respectively. Consequently, the reflection spectrum bandwidth of the chaotic BDG for the FOF scheme was a little narrower than that for the COF scheme. Figure 8(b) shows the gain spectra of the chaotic BDG before and after the elimination of the time-delay-induced spurious BDG. We can see that there was slight variation in the gain spectral width of the chaotic BDG, which was possibly related to the difference in the chaotic states before and after the utilization of the FOF. Therefore, the FOF not only can eliminate the spurious BDG but can also maintain the good reflection and gain spectra of the chaotic BDG.

## Conclusions

In summary, a theoretical model of the chaotic BDG for the COF and FOF schemes has been established using Lang-Kobayashi single-mode rate equations and SBS five-wave coupled equations. The FOF is a simple and effective feedback control method for suppressing the time-delay signatures of chaotic lasers. Under the proper filter parameters, i.e., the spectral width of the filter and its detuning from the laser frequency, a chaotic signal without time-delay signature can be generated and further utilized to form a chaotic BDG. By comparing the COF and FOF schemes, it was found that the time-delay-induced spurious BDG was effectively eliminated, and the reflection characterization of the chaotic BDG was improved. Optimized chaotic BDGs have potential for use in distributed fiber sensing, tunable optical delays and all-optical signal processing.

## Methods

For the generation and reading processes of the chaotic BDG in a PMF, a theoretical model is established using the following SBS five-wave coupled equations^25^:

5-1 $${\partial }_{z}{A}_{p1}+{\beta }_{1s}{\partial }_{t}{A}_{p1}=-\eta {g}_{B}Q{A}_{p2},$$

5-2 $$-\,{\partial }_{z}{A}_{p2}+{\beta }_{1s}{\partial }_{t}{A}_{p2}=\eta {g}_{B}{Q}^{\ast }{A}_{p1},$$

5-3 $$-\,{\partial }_{z}{A}_{p}+{\beta }_{1f}{\partial }_{t}{A}_{p}=\eta {g}_{B}{Q}^{\ast }{A}_{r}{e}^{i{\rm{\Delta }}kz},$$

5-4 $${\partial }_{z}{A}_{r}+{\beta }_{1f}{\partial }_{t}{A}_{r}=-\eta {g}_{B}Q{A}_{p}{e}^{-i{\rm{\Delta }}kz},$$

5-5 $${\partial }_{z}Q+(1/2{\tau }_{B}-i{\rm{\Delta }}\omega )Q=({A}_{p1}{A}_{p2}^{\ast }+{A}_{r}{A}_{p}^{\ast }{e}^{i{\rm{\Delta }}kz})/2{\tau }_{B}.$$

*A*_*p*1_, *A*_*p*2_, *A*_*p*_, and *A*_*r*_ are the slowly varying envelopes of the electric fields, i.e., the Pump1, Pump2, Probe and Reflection waves, respectively, and *Q* is the acoustic wave field induced by the electrostriction effect in the SBS process of Pump1 and Pump2. *β*_1*s*_ and *β*_1*f*_ are the group delays per unit length of the PMF slow and fast axes, respectively. *g*_*B*_ is the SBS gain coefficient, *τ*_*B*_ is the acoustic wave lifetime, and Δ*k* is the phase detuning which is directly related to the frequency difference Δ*υ*_*Bire*_ of the probe and Pump2. When the phase detuning Δ*k = *0, the frequency difference Δ*υ*_*Bire*_ = *υ*_probe_ − *υ*_pump2_ = Δ*nυ*_probe_/*n*, where Δ*n* = *n*_*x*_ − *n*_*y*_ is the PMF birefringence, and *n*_*x*_ and *n*_*y*_ are the refractive indexes of the slow and fast axes of the PMF, respectively. Δ*ω* = *ν*_pump1_ − *ν*_pump2_ − *ν*_*B*_ is the frequency detuning of Pump1 and Pump2. All the involved parameters and their values used in our theoretical model are from ref.^25^.

The dynamics of a semiconductor laser with FOF can be descripted using the Lang-Kobayashi single-mode rate equations as follows^29^:

6-1 $$\frac{dE(t)}{dt}=\frac{1}{2}(1+i\alpha )[G(t)-\frac{1}{{\tau }_{p}}]E(t)+\frac{\kappa }{{\tau }_{in}}F(t),$$

6-2 $$\frac{dF(t)}{dt}={\rm{\Lambda }}E(t-\tau ){e}^{-i\omega \tau }+(i{\rm{\Delta }}\nu -{\rm{\Lambda }})F(t),$$

6-3 $$\frac{dN(t)}{dt}=\frac{I}{qV}-\frac{N(t)}{{\tau }_{n}}-G(t){|E(t)|}^{2},$$

6-4 $$G(t)=\frac{g(N(t)-{N}_{0})}{1+\varepsilon {|E(t)|}^{2}}.$$

*E*, *N* and *F* are the slowly varying complex electrical field amplitude, the carrier density in the laser cavity and the complex field amplitude reentering from the laser cavity via the FOF, respectively. *α* is the line-width enhancement factor, and *g* is the differential gain coefficient. *τ*_*p*_ and *τ*_*n*_ are the photon and carrier lifetimes, respectively. *κ* is the feedback strength of the external cavity, *τ*_*in*_ is the round-trip time in the laser intracavity, and *τ* is the round-trip time delay of the external cavity, Λ and Δ*ν* are the spectral width of the filter and its detuning from the laser frequency, respectively. *ω* is the output angular frequency of the semiconductor laser, and *I* and *I*_th_ are the pump current and the threshold current, respectively. *q* is the charge quantity, *V* is the active region volume, *ε* is the gain saturation parameter, and *N*_0_ is the carrier density at transparency. All the involved laser parameter values of the semiconductor laser with FOF used in our numerical model refer to those in ref. 29.
